# Correlation between systolic blood pressure variability and in-hospital mortality in patients with acute aortic dissection

**DOI:** 10.3389/fcvm.2026.1868697

**Published:** 2026-08-14

**Authors:** Xinyi Chen, Jiahua Chen, Xinqun Li, Xueyan Li, Xiusong Wang

**Affiliations:** 1 Department of Emergency, The First Affiliated Hospital of Wenzhou Medical University, Wenzhou, China; 2 Department of Gynecology, The First Affiliated Hospital of Wenzhou Medical University, Wenzhou, China; 3 Department of Thoracic and Cardiovascular Surgery, The First Affiliated Hospital of Wenzhou Medical University, Wenzhou, China

**Keywords:** aortic dissection, average real variability, blood pressure variability, emergency, in-hospital mortality

## Abstract

**Background:**

High blood pressure variability (BPV) may be associated with in-hospital mortality in patients with acute aortic dissection(AAD). This study aimed to investigate the association between BPV and in-hospital mortality in patients with AAD.

**Methods:**

This retrospective study included patients with AAD who presented to the emergency department of the First Affiliated Hospital of Wenzhou Medical University between January 2024 and June 2025. In-hospital mortality was defined as the endpoint for in-hospital mortality. Patients with AAD were categorized into survivor and non-survivor groups according to in-hospital mortality. Blood pressure data during the emergency hospitalization period were collected, and BPV indices, including standard deviation (SD), coefficient of variation (CV), average real variability (ARV), as well as admission, mean, maximum, and minimum systolic and diastolic blood pressure values, were calculated. Multivariable logistic regression models were used to assess the associations.

**Results:**

A total of 216 patients with aortic dissection were included, of whom 27 died during hospitalization. Significant differences were observed in systolic blood pressure parameters, including admission, mean, and minimum values, as well as variability indices (SD, CV, VIM, and ARV) (all *P* < 0.05). Similarly, diastolic blood pressure parameters, including mean and minimum values and variability indices (ARV and VIM), differed significantly between the two groups (all *P* < 0.05). Multivariable logistic regression analysis demonstrated that SBP-SD and SBP-ARV were independently associated with in-hospital mortality in AAD (OR = 1.043, 95% CI: 1.002–1.085, *P* = 0.039; OR = 1.061, 95% CI: 1.002–1.123, *P* = 0.043).

**Conclusions:**

Elevated BPV was found to be associated with an increased risk of in-hospital mortality in patients with AAD. Among the BPV indices, systolic blood pressure ARV was observed to exhibit a relatively stronger association with in-hospital mortality. These findings suggest that BPV may serve as a potential risk marker for adverse in-hospital outcomes in AAD, thereby providing additional insight into blood pressure management strategies.

## 1 Introduction

Acute aortic dissection (AAD) is a life-threatening cardiovascular emergency associated with high morbidity and mortality (1). Despite significant advances in the treatment of AAD, the in-hospital mortality remains approximately 22% for Stanford type A AAD and approximately 13% for Stanford type B AAD (2, 3).

Blood pressure management is a cornerstone of initial AAD treatment, as controlling heart rate and blood pressure reduces aortic wall stress, while uncontrolled hypertension not only accelerates anatomical progression but also causes target organ damage, and the quality of blood pressure control is closely associated with patient prognosis (3–5).

Blood pressure variability (BPV) refers to the degree of fluctuation in blood pressure over time and represents an important indicator of blood pressure stability (6, 7). In recent years, BPV has increasingly been recognized as a novel risk factor for cardiovascular disease(CVD), providing a more accurate estimation of clinical outcomes in patients. However, the relationship between BPV and in-hospital mortality in patients with AAD remains unclear. Hence, this study aims to investigate the association between BPV and in-hospital mortality in patients with AAD.

## 2 Methods

### 2.1 Study population

The medical records of 216 patients with AAD at the Emergency Department of the First Affiliated Hospital of Wenzhou Medical University between January 2024 and June 2025 were retrospectively reviewed. The inclusion criteria were: (1) age ≥18 years and (2) diagnosis of AAD based on the results of aortic computed tomography angiography (CTA) (8). The exclusion criteria were as follows: (1) malignancy or other severe diseases; (2) pregnancy; and (3) incomplete medical records. In-hospital mortality was defined as the endpoint for in-hospital mortality. The research was conducted in accordance with the principle of the Declaration of Helsinki and approved by the Medical Ethics Committee established in the First Affiliated Hospital of Wenzhou Medical University, China (KY2026-R027).

### 2.2 BPV measurements

Upon admission, blood pressure was measured in both upper extremities, and the higher value was used as the reference value. Thereafter, blood pressure was recorded at 1-hour intervals. The parameters of blood pressure variability included the standard deviation (SD), coefficient of variation (CV), average real variability (ARV) (9), and variability independent of the mean (VIM) (10). We also measured admission, mean, maximum, and minimum SBP/DBP values. The CV was calculated as the standard deviation divided by the mean. ARV was defined as the average of the absolute differences between consecutive blood pressure measurements. The VIM is calculated first as the standard deviation of BP readings divided by the mean BP raised to the power of x, where×is obtained from fitting a non-linear regression model among the entire sample where standard deviation = a*mean^x^. This quantity is then multiplied by the sample mean BP raised to the power of x. All parameters were evaluated for both systolic blood pressure (SBP) and diastolic blood pressure (DBP). The ARV index, CV index and VIM index are calculated using the following formulas (10–12).

$$ARV=\frac{1}{\sum w}\times \overset{N-1}{\underset{k=1}{\sum }}\;w\times |BP_{k+1}-BP_{k}|$$

$$CV=\sqrt{\frac{\sum_{k=1}^{N}\;{\left(BP_{k}-\bar{BP}\right)}^{2}}{\left(N-1\right)}}/\bar{BP}$$

$$VIM=\frac{KxStandardDeviation(SBP)}{Mean{\left(SBP\right)}^{x}}$$

where, $K=Mean(Mean(SBP){)}^{x}$, *N* denotes the number of valid BP measurements in the data corresponding to a given subject, and *k* ranges from 1 to *N*-1. *W* means the interval time between two adjacent blood pressure records. In this study, ARV was used as the primary exposure variable because it can be relatively easily calculated in clinical practice and allows for the definition of a common reference framework.

### 1.1 Statistical analysis

Quantitative variables with a normal distribution were expressed as mean ± standard deviation ($\bar{X}$ ± SD) whereas non-normally distributed data were presented as [M (P25, P75)]. Depending on the data distribution, comparisons were performed using the t-test or the rank-sum test. Categorical variables, including patient characteristics, were compared using the chi-square test. Comparisons among multiple groups were conducted using analysis of variance (ANOVA) or the Kruskal–Wallis test. Logistic regression models were employed to assess the relationship between BPV and in-hospital mortality. Both unadjusted and multivariable-adjusted models were used. Variables included in the adjusted models were selected based on clinical relevance and results from univariate analyses (*P* < 0.05). Additionally, subgroup analyses were performed using logistic regression models, and interactions between subgroups were assessed using likelihood ratio tests. All analyses were performed using R Statistical Program (http://www.R-project.org, The R Foundation) and SPSS statistical software (version 22.0). *P*-value < 0.05 was considered statistically significant.

## 3 Results

### 3.1 Study population

Among the 216 patients included in the study, the mean age was 53.23 ± 13.05 years, with 82.4% being male. Of these patients, 83.8% underwent surgical intervention, and 27 (12.5%) died during hospitalization. Patients were stratified into quartiles based on systolic and diastolic blood pressure ARV. Table 1 summarizes the baseline characteristics of the study population across ARV quartiles. For systolic ARV, quartiles were defined as Q1 (≤ 8.03, *n* = 54), Q2 (8.04–11.61, *n* = 54), Q3 (11.62–16.2, *n* = 54), and Q4 (> 16.2, *n* = 54), with significant intergroup differences observed in sex, type, treatment, length of hospital stay, mortality, and white blood cell count. For diastolic ARV, quartiles were defined as Q1 (≤ 5, *n* = 55), Q2 (5.01–7.27, *n* = 54), Q3 (7.28–10.3, *n* = 53), and Q4 (>10.3, *n* = 54), with significant differences in mortality, white blood cell count, lymphocyte count, and lactate levels.

**Table 1:** Association between average real variability and in-hospital mortality.

Variable	Quartiles of ARV of Systolic BP					Quartiles of ARV of Diastolic BP				
	1st quartile (*n* = 54)	2nd quartile (*n* = 54)	3rd quartile (*n* = 54)	4th quartile (*n* = 54)	*P*-value	1st quartile (*n* = 55)	2nd quartile (*n* = 54)	3rd quartile (*n* = 53)	4th quartile (*n* = 54)	*P*-value
Range	≤8.03	8.04–11.61	11.62–16.2	>16.2		≤5	5.01–7.27	7.28–10.3	>10.3	
Type, *n* (%)					0.02					0.304
Stanford A	38 (70.4)	24 (44.4)	27 (50)	35 (64.8)		33 (66)	28 (51.9)	27 (50.9)	36 (66.7)	
Stanford B	16 (29.6)	30 (55.6)	27 (50)	19 (35.2)						
Treatment, *n* (%)					0.004					0.096
Surgery	50 (92.6)	46 (85.2)	48 (88.9)	37 (68.5)		50 (90.9)	48 (88.9)	42 (79.2)	41 (75.9)	
Non-surgical	4 (7.4)	8 (14.8)	6 (11.1)	17 (31.5)		5 (9.1)	6 (11.1)	11 (20.8)	13 (24.1)	
Age, year	51.44 ± 12.813	53.24 ± 12.885	53.87 ± 13.172	54.35 ± 13.477	0.676	53.67 ± 12.14	52.96 ± 11.87	54.15 ± 14.31	52.13 ± 14.01	0.867
Male, *n* (%)	48 (88.9)	38 (70.4)	44 (81.5)	48 (88.9)	0.036	45 (81.8)	45 (83.3)	41 (77.4)	47 (87)	0.62
Heart rate on arrival, beats/min	70.91 ± 13.98	74.82 ± 18.4	78 ± 16.52	78.2 ± 18.04	0.085	72.22 ± 15.38	76.52 ± 14.86	74.28 ± 17.88	78.94 ± 19.15	0.919
Hypertension, *n* (%)	37 (68.5)	45 (83.3)	44 (81.5)	48 (88.9)	0.053	43 (78.2)	42 (77.8)	43 (81.1)	46 (85.2)	0.75
Diabetes, *n* (%)	3 (5.6)	4 (7.4)	2 (3.7)	3 (5.6)	0.872	4 (7.3)	2 (3.7)	4 (7.5)	2 (3.7)	0.702
Smoker/Drinker, *n* (%)	4 (7.4)	1 (1.9)	1 (1.9)	3 (5.6)	0.372	2 (3.6)	2 (3.7)	2 (3.8)	3 (5.6)	0.951
Hydrothorax, *n* (%)	8 (14.8)	12 (22.2)	12 (22.2)	6 (11.1)	0.327	8 (14.5)	13 (24.1)	5 (9.4)	12 (22.2)	0.161
Hydropericardium, *n* (%)	7 (13)	6 (11.1)	10 (18.5)	14 (25.9)	0.168	9 (16.4)	7 (13)	8 (15.1)	13 (24.1)	0.445
WBC,×109/L	11.25 ± 3.6	12.76 ± 4.56	12.99 ± 4.08	13.88 ± 5.49	0.24	12.04 ± 4.11	12.82 ± 3.55	11.51 ± 4.25	14.5 ± 5.6	0.004
NEU,×109/L	9.38 ± 3.71	10.81 ± 4.2	10.99 ± 4.17	11.31 ± 5.61	0.119	9.96 ± 3.91	10.79 ± 3.92	9.34 ± 4.48	12.39 ± 5.15	0.003
LYM,×109/L	1.02 (0.65,1.33)	1.07 (0.74,1.5)	0.98 (0.72,1.32)	1.1 (0.74,1.74)	0.241	1.05 (0.75,1.51)	0.99 (0.64,1.23)	0.99 (0.68,1.46)	1.13 (1.74,1.68)	0.205
RBC,×109/L	4.39 ± 0.65	4.38 ± 0.55	4.4 ± 0.61	4.48 ± 0.61	0.854	4.42 ± 0.61	4.43 ± 0.54	4.37 ± 0.6	4.45 ± 0.66	0.916
Hemoglobin, g/L	132.06 ± 20.55	133.41 ± 18.89	135.61 ± 18.55	137.05 ± 18.43	0.534	134.8 ± 20.22	134.29 ± 15.43	133.04 ± 19.11	135.96 ± 21.46	0.887
Platelet×109/L	171.76 ± 44.6	195.06 ± 65.39	183.02 ± 54.47	174.87 ± 55.38	0.131	179.89 ± 53.46	182.48 ± 47	178.25 ± 65.77	184.07 ± 56.95	0.951
Lactate, mmol/L	1.55 (0.98,2.43)	2.1 (1.1,3.1)	2.1 (1.1,2.8)	2.25 (1.2,3.7)	0.129	1.8 (1,2.4)	2 (1.1,2.85)	1.6 (1.1,2.6)	2.7 (1.2,4.23)	0.012
Blood glucose, mmol/L	7.05 (5.98,8.2)	7.65 (6.88,8.55)	7.7 (6.55,8.7)	8.15 (7.1,10)	0.007	7.5 (6.4,8.4)	7.9 (6.1,8.7)	7.4 (6.7,8.7)	7.75 (7.05,10.8)	0.254
BUN, mmol/L	5.95 (4.48,8.05)	5.9 (4.75,7.35)	5.75 (4.7,6.9)	6.25 (5.1,7.1)	0.637	6.1 (4.9,8.1)	6.1 (4.78,7.5)	5.6 (4.5,6.65)	6.15 (4.9,6.93)	0.16
CK-MB, ng/mL	1.63 (1.09,2.47)	1.88 (1.09,3.66)	2.22 (1.41,3.63)	2.27 (1.68,3.6)	0.033	1.79 (1.25,2.83)	2.2 (1.39,3.63)	1.94 (1.28,2.89)	2.02 (1.27,3.66)	0.8029
Hospital stay, day	21.02 ± 8.875	19.56 ± 9.197	19.43 ± 8.077	14.75 ± 10.546	0.004	21.05 ± 8.49	19.35 ± 9.01	17.65 ± 8.75	16.69 ± 10.97	0.078
Non-survivor, *n* (%)	3(5.6)	4(7.4)	5(9.3)	15(27.8)	0.001	4(7.3)	3(5.6)	8(15.1)	12(22.2)	0.033

### 1.2 Analysis of blood pressure variability between survivors and non-survivors

**Figure 1:**
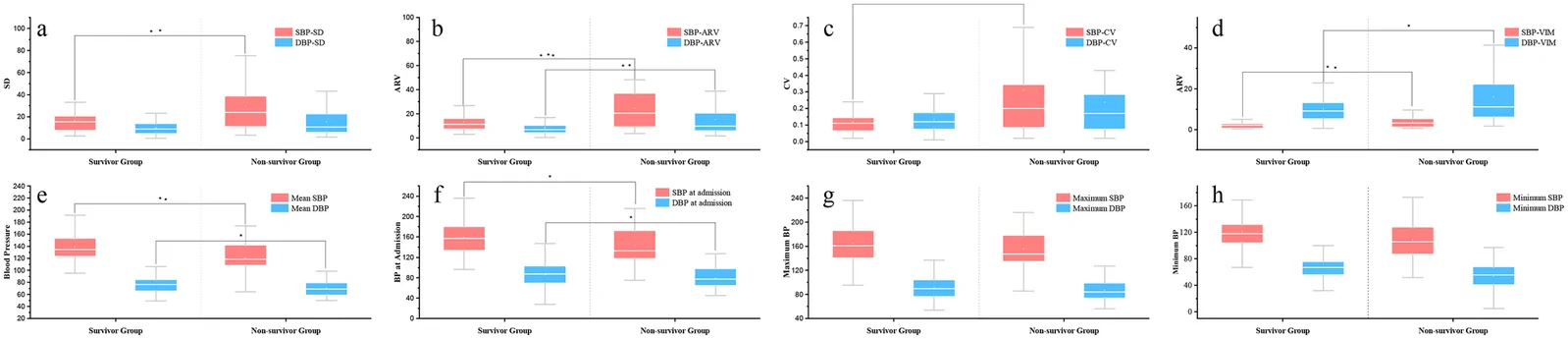
Comparison of BPV between the non-survival group and the survival group. * *P* < 0.05, ** *P* < 0.01, *** *P* < 0.001.

Blood pressure variability indices were compared between deceased and surviving AAD patients. Significant differences were observed in systolic blood pressure parameters, including admission, mean, minimum, SD, CV, VIM, and ARV (all *P* < 0.05). Similarly, diastolic blood pressure parameters, including mean, minimum, ARV, and VIM, also differed significantly between the two groups (all *P* < 0.05), as detailed in Table 2 and Figure 1.

**Table 2:** Differences in BPV between patients in the survivor and Non-survivor groups.

Variables	Survivor (*n* = 189)	Non-survivor (*n* = 27)	t/z-value	*P*-value
SBP at admission	158.97 ± 32.398	142.26 ± 38.209	2.449	0.015
Mean SBP	138.88 ± 22.75	120.44 ± 32.65	−2.838	0.008
Maximum SBP	161 (142,185)	147 (136,177)	−1.416	0.157
Minimum SBP	118 (105,131)	106 (88,127)	−2.413	0.016
SBP-SD	15.37 (8.37,20.12)	24.01 (11.79,38)	−3.244	0.001
SBP-CV	0.11 (0.07,0.15)	0.2 (0.09,0.34)	−3.643	<0.001
SBP-ARV	11.27 (8,15.5)	20.5 (9.88,36.5)	−3.493	<0.001
SBP-VIM	2.016 (1.091,2.638)	3.114 (1.549,4.998)	−3.198	0.001
DBP at admission	85.88 ± 22.901	78.63 ± 20.866	1.555	0.122
Mean DBP	76.14 ± 13.68	70.45 ± 14.31	−2.011	0.046
Maximum DBP	90 (78,103)	84 (75,98)	−1.185	0.236
Minimum DBP	66.68 ± 14.19	53.26 ± 24.549	2.776	0.01
DBP-SD	8.85 (5.51,13.39)	10.61 (6.51,21.93)	−1.666	0.096
DBP-CV	0.12 (0.08,0.17)	0.16 (0.08,0.28)	−1.924	0.054
DBP-ARV	7 (5,9.92)	9.79 (6.5,20)	−2.816	0.005
DBP-VIM	9.043 (5.554,12.891)	11.138 (6.441,21.882)	−1.796	0.073

### 1.3 Blood pressure variability and in-hospital mortality

**Figure 2:**
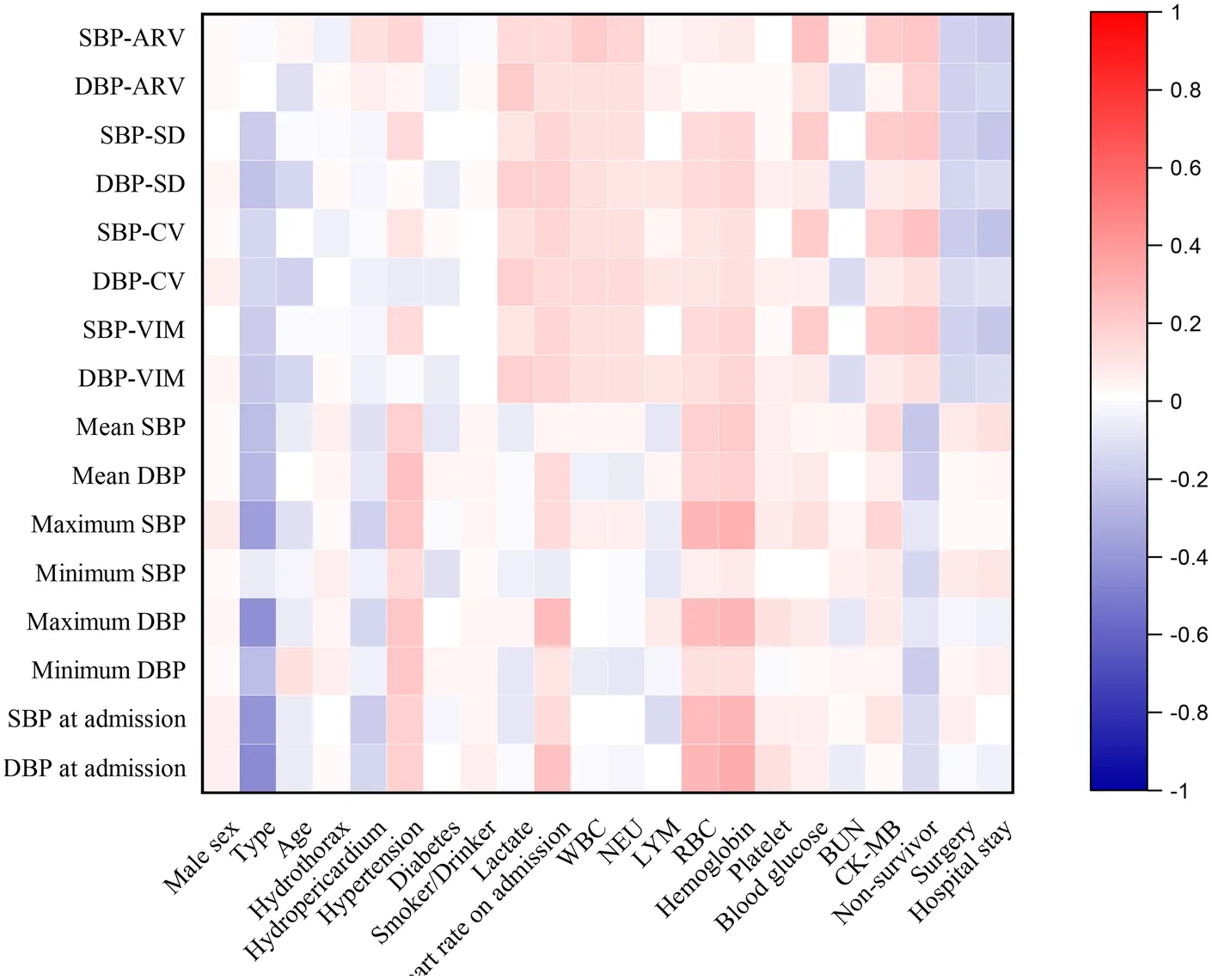
Correlation plot of the associations of BPV with general characteristics,laboratory investigations and prognosis. The color gradient from blue to red corresponds to the correlation coefficient, ranging from −1 to +1.A coefficient of 0 indicates no correlation. WBC, white blood cell. NEU, neutrophil. LYM, lymphocyte. RBC, red blood cell. BUN, blood urea nitrogen. CK-MB, creatine kinase-MB.

A correlation heatmap was constructed to illustrate the relationship between blood pressure variability and early prognosis in patients with AAD (Figure 2). Spearman rank correlation analysis was performed to assess the associations between blood pressure variability indices and in-hospital mortality in AAD. For systolic blood pressure, SD, CV, ARV and VIM were positively correlated with in-hospital mortality. Similarly, SD and CV of diastolic blood pressure showed positive correlations (all *P* < 0.05). In contrast, mean, admission, and minimum systolic blood pressure, as well as admission and minimum diastolic blood pressure, were negatively correlated with in-hospital mortality in patients with AAD (Table 3).

**Table 3:** Spearman correlation analysis of BPV compared with in-hospital mortality.

Variables	*ρ*	*P*-value
SBP at admission	−0.15	0.028
Mean SBP	−0.208	0.002
Maximum SBP	−0.097	0.157
Minimum SBP	−0.165	0.015
SBP-SD	0.221	0.001
SBP-CV	0.248	<0.001
SBP-ARV	0.238	<0.001
SBP-VIM	0.218	0.001
DBP at admission	−0.137	0.045
Mean DBP	−0.169	0.13
Maximum DBP	−0.081	0.237
Minimum DBP	−0.204	0.003
DBP-SD	0.114	0.096
DBP-CV	0.131	0.054
DBP-ARV	0.192	0.005
DBP-VIM	0.122	0.072

Table 4 summarizes the odds ratios (ORs) and 95% confidence intervals (CIs) for the associations between BPV and in-hospital mortality. Based on the results presented in Table 5 and their clinical relevance, age, type, gender, treatment, hydropericardium, RBC, hemoglobin, lactate, blood glucose, CK-MB were included in the multivariable logistic regression analysis. Each BPV parameter was independently incorporated into the logistic regression models. Multivariable logistic regression analyses were performed to evaluate the associations between BPV parameters and adverse outcomes across different models. In the unadjusted model, except maximum SBP, DBP at admission, and maximum DBP, were associated with increased in-hospital mortality. In Model 2, after adjustment for gender, age, type, and treatment, SBP-SD, SBP-ARV, SBP-VIM, and DBP-ARV remained positively associated with in-hospital mortality. In Model 3, the associations of SBP-SD and SBP-ARV with in-hospital mortality remained statistically significant (OR = 1.043, 95% CI: 1.002–1.085, *P* = 0.039; OR = 1.061, 95% CI: 1.002–1.123, *P* = 0.043). However, DBP-ARV was not significantly associated with in-hospital mortality (OR = 1.066, 95% CI: 0.965–1.178, *P* = 0.209).

**Table 4:** Logistic regression models evaluating the association of BPV predictors with in-hospital mortality.

Variable	Model1			Model2			Model3		
	*P*-value	OR	95% C.I.	*P*-value	OR	95% C.I.	*P*-value	OR	95% C.I.
SBP at admission	0.017	0.985	0.972–0.997	0.98	1.001	0.983–1.018	0.171	1.015	0.994–1.036
Mean SBP	<0.001	0.967	0.949–0.985	0.373	0.99	0.967–1.013	0.363	0.989	0.966–1.013
Maximum SBP	0.161	0.99	0.976–1.004	0.639	1.005	0.985–1.025	0.08	1.022	0.997–1.046
Minimum SBP	0.012	0.975	0.956–0.994	0.185	0.983	0.959–1.008	0.955	0.999	0.974–1.026
SBP-SD	<0.001	1.055	1.026–1.085	0.043	1.038	1.001–1.077	0.039	1.043	1.002–1.085
SBP-CV	0.002	730.402	10.463–50,988.662	0.06	55.544	0.848–3,638.014	0.061	53.318	0.836–3,402.56
SBP-ARV	<0.001	1.078	1.04–1.117	0.014	1.061	1.012–1.112	0.043	1.061	1.002–1.123
SBP-VIM	<0.001	1.503	1.212–1.863	0.043	1.334	1.01–1.763	0.067	1.001	0.999–1.001
DBP at admission	0.123	0.986	0.969–1.004	0.594	1.008	0.979–1.038	0.266	1.02	0.985–1.057
Mean DBP	0.047	0.968	0.938–1	0.706	0.992	0.952–1.034	0.681	0.991	0.95–1.034
Maximum DBP	0.272	0.988	0.966–1.01	0.576	1.01	0.976–1.044	0.435	1.017	0.975–1.06
Minimum DBP	<0.001	0.954	0.929–0.978	0.257	0.979	0.943–1.016	0.498	0.986	0.945–1.028
DBP-SD	0.003	1.075	1.026–1.127	0.081	1.086	0.99–1.192	0.254	1.064	0.956–1.185
DBP-CV	0.001	217.147	9.249–5,097.978	0.057	336.711	0.841–1,34,835.424	0.216	64.718	0.088–47,666.515
DBP-ARV	<0.001	1.101	1.048–1.157	0.042	1.089	1.003–1.183	0.209	1.066	0.965–1.178
DBP-VIM	0.001	1.078	1.029–1.129	0.072	1.088	0.992–1.193	0.244	1.064	0.959–1.18

Model 1: Unadjusted.

Model 2: Adjusted for gender, age, type, treatment.

Model 3: Adjusted for model 2 plus variables including hydropericardium, RBC, hemoglobin, lactate, blood glucose and CK-MB.

**Table 5:** Background characteristics of study participants (*n* = 216).

Variables	Survivor (*n* = 189)	Non-survivor (*n* = 27)	*P*-value
Type, *n* (%)			<0.001
Stanford A	98 (51.9)	26 (96.3)	
Stanford B	91 (48.1)	1 (3.7)	
Treatment, *n* (%)			<0.001
Surgery	171 (90.5)	10 (37)	
Non-surgical	18 (9.5)	17 (63)	
General characteristic			
Age, year	52.63 ± 12.502	57.41 ± 16.005	0.075
Male, *n* (%)	155 (82)	23 (85.2)	0.685
Hypertension, *n* (%)	150 (79.4)	24 (88.9)	0.242
Diabetes, *n* (%)	11 (5.8)	1 (3.7)	0.653
Smoker/Drinker, *n* (%)	7 (3.7)	2 (7.4)	0.368
Presenting symptoms, *n* (%)			
Chest pain	17 (63)	140 (74.1)	0.225
Back pain	77 (40.7)	6 (22.2)	0.064
Chest and back pain	53 (28)	4 (14.8)	0.145
Syncope	4 (2.1)	1 (3.7)	0.608
Abdominal and lumbar pain	48 (25.4)	6 (22.2)	0.722
Diaphoresis	6 (3.2)	3 (11.1)	0.054
Nausea and vomiting	4 (2.1)	1 (3.7)	0.608
Chest tightness	29 (15.3)	7 (25.9)	0.168
Weakness	5 (2.6)	2 (7.4)	0.191
Laboratory investigations			
Hydrothorax, *n* (%)	34 (18)	4 (14.8)	0.685
Hydropericardium, *n* (%)	27 (14.3)	10 (37)	0.003
WBC,×109/L	12.511 ± 4.269	14.173 ± 6.102	0.076
NEU,×109/L	10.45 ± 4.315	11.84 ± 5.631	0.134
LYM,×109/L	1 (0.7，1.415)	1.24 (0.89，.98)	0.079
RBC,×109/L	4.45 ± 0.576	4.157 ± 0.719	0.017
Hemoglobin,g/L	135.606 ± 18.955	127.002 ± 18.624	0.028
Platelet×109/L	183.854 ± 55.105	162.458 ± 58.058	0.062
Lactate,mmol/L	1.8 (1.1,2.65)	3 (1.3,7.4)	0.003
Blood glucose,mmol/L	7.6 (6.7，8.55)	8.5 (6.9，1.4)	0.017
BUN,mmol/L	5.9 (4.7，6.9)	7.1 (6.5，8.0)	<0.001
CK-MB,ng/mL	1.86 (1.265，3.11)	2.39 (1.46，10.1)	0.013

### 1.4 Stratified analyses

Stratified analyses were performed according to treatment strategy, aortic dissection type, gender, and age to assess the associations between SBP-SD and SBP-ARV and in-hospital mortality across subgroups. No significant interactions were observed between subgroups with respect to in-hospital mortality (*P* for interaction > 0.05), as detailed in Tables 6, 7.

**Table 6:** Odds ratio of adverse outcomes associated with SBP-SD, by subgroup.

Subgroup	Category	Count	Percent	OR (95% C.I.)	*P*-value	*P* for interaction
Overall		216	100	1.05 (1.03,1.08)		
Treatment						0.383
	Surgery	181	83.8	1.056 (0.981–1.071)	0.273	
	Non-surgical	35	16.2	1.025 (1.004–1.111)	0.034	
Type						0.306
	Stanford B	92	42.6	1.18 (0.96, 1.4	0.116	
	Stanford A	124	57.4	1.06 (1.02,1.09)	0.001	
Gender						0.083
	Female	38	17.6	1.19 (1.03, 1.37)	0.017	
	Male	178	82.4	1.05 (1.02, 1.07)	0.001	
Age						0.181
	>60	55	25.5	1.11 (1.03,1.2)	0.008	
	≤ 60	161	74.5	1.05 (1.02,1.08)	0.001	

**Table 7:** Odds ratio of adverse outcomes associated with SBP-ARV, by subgroup.

Subgroup	Category	Count	Percent	OR (95% C.I.)	*P*-value	*P* for interaction
Overall		216	100	1.05 (1.03,1.08)		
Treatment						0.938
	Surgery	35	16.2	1.07 (1.01, 1.14)	0.045	
	Non-surgical	181	83.8	1.07 (1.01, 1.12)	0.018	
Type						0.329
	Stanford B	92	42.6	1.18 (0.97, 1.43)	0.096	
	Stanford A	124	57.4	1.07 (1.03, 1.11)	0.001	
Gender						0.781
	Female	38	17.6	1.1 (0.96, 1.25)	0.161	
	Male	178	82.4	1.08 (1.04, 1.12)	＜0.001	
Age						0.791
	>60	55	25.5	1.1 (1, 1.21)	0.059	
	≤ 60	161	74.5	1.08 (1.04, 1.13)	0.001	

## 4 Discussion

Hypertension is a key risk factor for AAD, and blood pressure has been shown to be positively associated with both the incidence and mortality of the disease (13); therefore, blood pressure control is considered the cornerstone of medical management in AAD. The fundamental principle of aortic dissection management is to limit disease progression through blood pressure control and reduction of aortic wall stress (14). BPV is defined as fluctuations in blood pressure over a specified period and reflects complex physiological mechanisms (15). Previous studies have demonstrated that BPV is associated with outcomes in cardiovascular diseases such as stroke (16), heart failure (17), and myocardial infarction (18); however, its prognostic significance in AAD remains unclear.

In the present study, increased BPV in patients with AAD, particularly SBP-ARV, was found to be significantly associated with in-hospital mortality (*P*<0.05). Song et al. reported that high BPV has been identified as an independent risk factor for AAD (19), consistent with the present findings. However, prior research has primarily focused on hypertension (20) and diastolic blood pressure levels (21), with limited attention given to the prognostic role of BPV, particularly in the emergency setting (22).

Early studies have demonstrated an association between BPV and target organ damage (7, 23). The mechanisms linking BPV to target organ damage may involve increased arterial stiffness, microvascular remodeling, as well as activation of inflammatory cascades and neurohormonal systems (24). BPV may reflect alterations in autonomic nervous system activity, with greater variability indicating impaired autonomic regulation and exerting significant effects on vascular tone and cardiac load (25, 26). Elevated BPV may adversely affect hemodynamics, leading to arterial wall injury and accelerated atherosclerosis (27). In AAD, elevated BPV may exacerbate aortic injury, while autonomic instability may promote fluctuations in vascular tone, thereby increasing the risk of rupture or further dissection (28), ultimately contributing to adverse outcomes.

It has been shown that variability independent of the mean (VIM) is significantly associated with cardiovascular outcomes (10, 29). Although this study represents the first evaluation of VIM in patients with AAD, no independent association with in-hospital mortality was observed after multivariable adjustment. This discrepancy may reflect fundamental differences in study populations, as prior research has primarily focused on community-based cohorts with chronic atherosclerotic progression (10, 29), whereas AAD is characterized by acute pathology, with mortality predominantly driven by mechanical complications such as false lumen rupture and cardiac tamponade (22). In addition, the utility of VIM—which is designed to remove the influence of mean blood pressure—may be limited in the acute phase due to marked hemodynamic instability. The relatively small sample size and limited number of events may have further reduced the statistical power of the analysis. Moreover, the effect size of VIM may be smaller than those of SD or ARV, thereby requiring larger cohorts to achieve statistical significance.

Diastolic blood pressure variability has been widely associated with cardiovascular outcomes across diverse populations. It has been demonstrated that diastolic BPV independently predicts cardiovascular and all-cause mortality, and may, in some cases, provide greater prognostic value than systolic BPV (30–32). This association has been further supported by large-scale cohort analyses and meta-analyses (33) However, in the present study, multivariable analysis in Model 3 demonstrated that diastolic BPV indices (CV, SD, VIM, and ARV) were not independently associated with in-hospital mortality in patients with AAD. The underlying mechanisms remain unclear and may reflect differences in pathophysiological context. Compared with diastolic BPV, systolic BPV has been more strongly associated with arterial stiffness and acute hemodynamic stress (34). Previous studies have primarily focused on stable hypertensive populations with long-term follow-up, whereas acute aortic disease is characterized by early mortality predominantly driven by acute mechanical complications rather than cumulative vascular injury. In addition, the relatively small sample size may have limited the statistical power to detect modest effects. Therefore, these findings should be interpreted as context-specific rather than conclusive. Further large-scale studies with longer follow-up are warranted to elucidate the role of diastolic BPV in the prognosis of aortic dissection.

However, several limitations should be acknowledged in this study. First, this study was conducted as a retrospective, single-center analysis with a relatively small sample size, which may have compromised statistical robustness. In addition, all participants were of Chinese ethnicity; therefore, caution should be exercised when generalizing these findings to other populations or ethnic groups. Second, detailed information regarding surgical strategies, perioperative blood pressure management, and postoperative complications was not available. Furthermore, specific data on antihypertensive therapy during hospitalization were not available. These unmeasured confounders may have influenced both blood pressure measurements and clinical outcomes. Therefore, the present findings should be considered exploratory and warrant validation in larger, multicenter prospective studies. Nevertheless, increased systolic blood pressure variability may be associated with a higher risk of in-hospital mortality in patients with AAD, thereby providing a basis for future hypothesis generation and research directions.

## 5 Conclusion

Elevated BPV was found to be associated with an increased risk of in-hospital mortality in patients with AAD. Among the BPV indices, systolic blood pressure ARV was observed to exhibit a relatively stronger association with in-hospital mortality. These findings suggest that BPV may serve as a potential risk marker for adverse in-hospital outcomes in AAD, thereby providing additional insight into blood pressure management strategies.

## Data Availability

The original contributions presented in the study are included in the article/Supplementary Material, further inquiries can be directed to the corresponding authors.
